# Targeting dipeptidyl peptidase 3 (DPP3) in extreme‐critically ill patients with refractory shock: First‐in‐human report on the safety and efficacy of an anti‐DPP3 antibody

**DOI:** 10.1002/ejhf.3718

**Published:** 2025-07-10

**Authors:** Dominik Jarczak, Axel Nierhaus, Alexandre Mebazaa, Antoine Herpain, Peter Pickkers, Sanchir Anar, Stefan Kluge

**Affiliations:** ^1^ Department of Intensive Care Medicine University Medical Center Hamburg‐Eppendorf 20251 Hamburg Germany; ^2^ Université Paris Cité INSERM UMR‐S 942 (MASCOT), Paris France; ^3^ Department of Anesthesiology and Critical Care and Burn Unit, Saint‐Louis and Lariboisière Hospitals, FHU PROMICE, DMU Parabol, APHP Nord Paris France; ^4^ Department of Intensive Care, Saint‐Pierre University Hospital Université Libre de Bruxelles (ULB) Brussels Belgium; ^5^ Department of Intensive Care Radboud University Medical Centre PO Box 9101, NL 6500 HB Nijmegen The Netherlands

Refractory shock originates from severely depressed myocardial contractility – mostly due to septic shock, septic cardiomyopathy, sepsis‐induced vascular dysfunction and vascular hyporesponsiveness to vasoconstrictors (vasoplegia), acute myocardial infarction (AMI), or acute heart failure. It thereby initiates progressive worsening of systolic and diastolic dysfunction and results in systemic hypoperfusion, coronary ischaemia, increased ventricular filling pressures, multi‐organ system failure, and often death.^1^, ^2^ Moreover, this leads to inflammatory activation, anaerobic metabolism, and oxidative stress, resulting in systemic inflammatory response syndrome in one out of three patients.^3^

While specific therapies for refractory shock are missing, just recently dipeptidyl peptidase 3 (DPP3) has been validated as a druggable biotarget: DPP3 is an intracellular aminopeptidase and is released upon cell death – like in sepsis and AMI and degrades angiotensin II (Ang2) and related peptides, disrupts the renin–angiotensin–aldosterone system (RAAS) and thereby perpetuates shock.^4^, ^5^ Recently, a humanized monoclonal antibody that binds DPP3 and inhibits its enzymatic activity, has entered clinical evaluation. This anti‐DPP3 antibody was designed to block the enzymatic activity of circulating DPP3 in the bloodstream, thereby inhibiting DPP3‐dependent angiotensin peptide degradation and restoring RAAS homeostasis. This blockade is intended to rapidly stabilize haemodynamics and therefore cardiovascular and renal functions, improving clinical outcomes in affected patients. It showed excellent pre‐clinical safety and efficacy in mice, rats, pigs and monkeys, including normalization of cardiovascular, pulmonary and renal function, with drastically reduced mortality.^6^ A phase 1 study in healthy subjects (NCT06331884) was recently completed.

Here we report the treatment of three terminally critically ill patients with refractory shock and multi‐organ failure due to septic cardiomyopathy, using the anti‐DPP3 antibody. The treatment was conducted in the Department of Intensive Care Medicine, at the University Medical Center Hamburg‐Eppendorf in Hamburg, Germany. Patients were eligible, if they were (i) in refractory shock with continuous clinical deterioration, (ii) in utmost critical condition with signs of multi‐organ failure, and (iii) upon exhaustion of all therapeutic options. The treatment on a named‐patient basis approach relies on article 37 of the Declaration of Helsinki and article 41 of the German Pharmaceuticals Act.^7^ Accordingly, while approvals by the ethics committee and regulatory bodies are not applicable for this approach, informed consent was obtained from the patients, their relatives, or legal representatives.

Lactate, C‐reactive protein and interleukin‐6 were determined in the routine lab. DPP3 activity was measured using a customized assay. Follow‐up was pre‐specified as 48 h, with efficacy read‐out defined as shock reversal, indicated by a norepinephrine dose ≤0.2 μg/kg body weight/min or halving of initial dose.

Two female patients (patients 1 and 3) and one male patient (patient 2) were treated. Detailed information on patient characteristics and concomitant therapy are provided in online supplementary *Tables* *S1*–*S3*. The patients' age ranged between 64 and 84 years. All three patients had pre‐existing conditions and were in utmost critical condition with signs of multi‐organ failure, including high plasma lactate level, due to refractory septic shock. In addition, patient 1 had an AMI, which could not be immediately revascularized due to severe haemodynamic instability, while patient 3 was suffering from right‐sided cardiogenic shock. All three were suffering from acute kidney injury, in need of renal replacement therapy, two out of three (patients 1 and 2) were intubated and under invasive mechanical ventilation. Activity of DPP3 was strongly elevated in all three patients (≥99th percentile of a general population) (*Figure* *1*). All three patients were approved for treatment with the anti‐DPP3 antibody by an independent international patient selection board. The board confirmed exhaustion of all conventional therapeutic options with no further options for escalation of conventional therapy in the presence of a high chance of immediate mortality.

**Figure 1:**
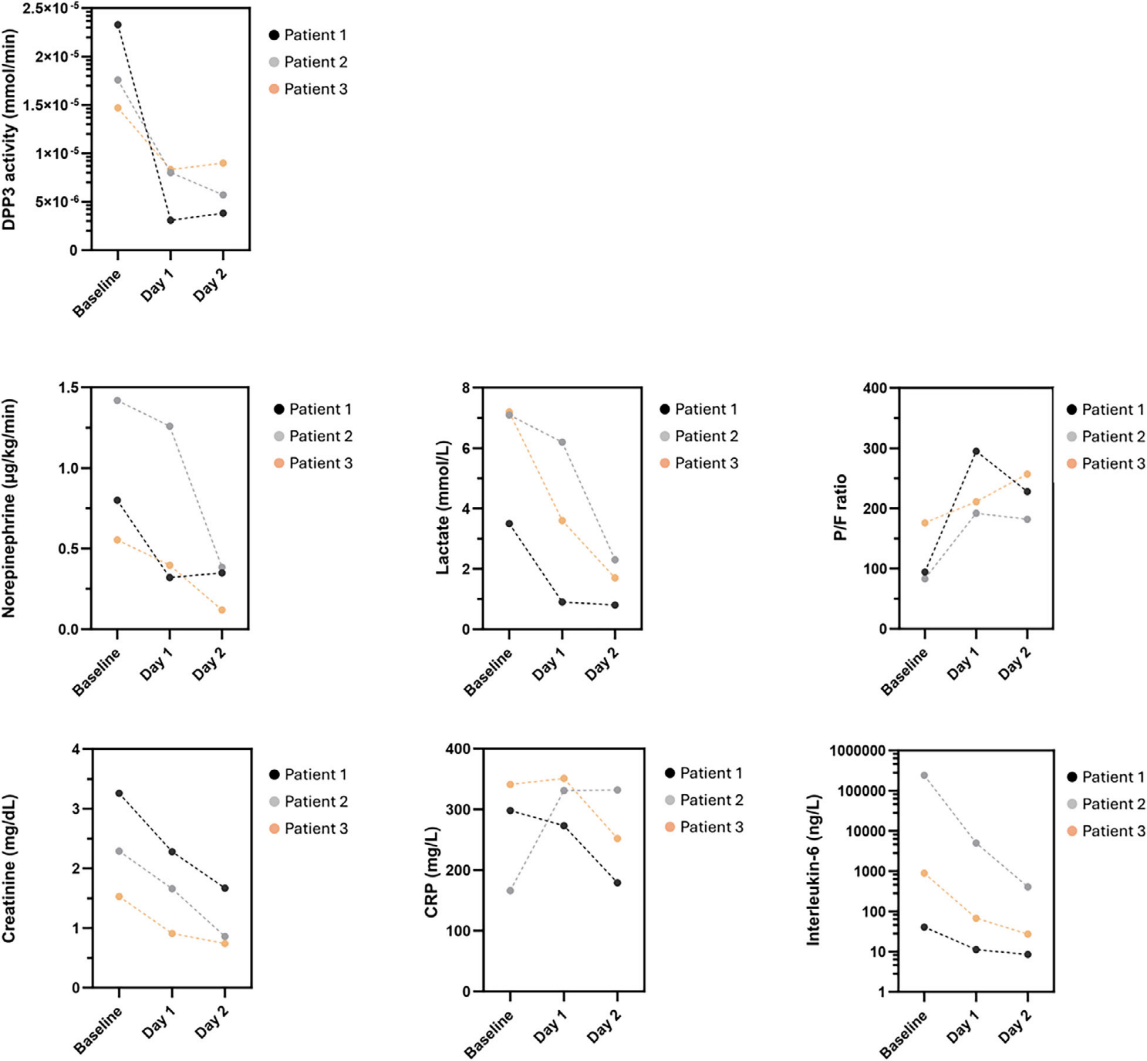
Effect of the anti‐dipeptidyl peptidase 3 (DPP3) antibody on DPP3 activity (upper panel), norepinephrine infusion rate, lactate concentration, and respiratory function (mid panel), renal function and inflammatory markers (lower panel). CRP, C‐reactive protein; P/F, ratio of partial pressure of oxygen in arterial blood to the fraction of inspiratory oxygen concentration.

Patients received a single dose of the anti‐DPP3 antibody at a dose of 10 mg/kg body weight over a 2‐h period. The anti‐DPP3 antibody was administered between 1 and 3 days after admission to the intensive care unit, separately from any concomitant drugs using a dedicated lumen of a central venous catheter. All patients were on stable medication at time of anti‐DPP3 antibody dosing and thereafter, and the antibody remained the only novel therapeutic intervention introduced during the 48‐h follow‐up period.

The therapy was well tolerated in all three patients, no immediate adverse reactions were noted, and no adverse events judged to be associated with the administration of anti‐DPP3 antibody. *Figure* *1* shows the effect of the intervention on DPP3 activity, norepinephrine infusion rate and lactate concentration. The mode‐of‐action observations from animal models were confirmed, as DPP3 enzyme activity was strongly reduced upon anti‐DPP3 antibody administration (reduction of median DPP3 activity from 7 μmol/min*L at baseline to 2 μmol/min*L at day 2). The anti‐DPP3 antibody demonstrated therapeutic efficacy in all clinical parameters investigated: the reduction of DPP3 activity was mirrored by a strong decrease of norepinephrine need and shock reversal in all three patients, as well as normalization of lactate concentrations within the 48‐h follow‐up period. There was also an improvement in respiratory and kidney function during follow‐up, as indicated by clearly improved P/F ratio and decreasing creatinine levels in all three patients (*Figure* *1*). Finally, there was a profound decrease in circulating interleukin‐6 from median 893.5 ng/L (upper limit of normal 4.4 ng/L) to 27.2 ng/L, in line with previous observations from animal studies.^6^ In line, median C‐reactive protein values decreased from 298 mg/L (upper limit of normal <5 mg/L) to 179 mg/L.

While follow‐up was pre‐specified as 48 h, mid‐term follow‐up was assessed retrospectively and is provided in online supplementary *Tables* *S1*–*S3*.

This first experience on DPP3‐based biotherapy indicates that the specific humanized monoclonal anti‐DPP3 antibody holds great promise for treatment of refractory shock and the findings are in line with the proposed mode‐of‐action: the specific inhibition of DPP3 enzyme activity restores RAAS homeostasis and thereby prevents Ang2 degradation, resulting in reducing norepinephrine doses and increased lactate clearance through improved perfusion. This restoration of perfusion and circulatory function may improve renal function, as evidenced by decreased creatinine concentrations. We assume that besides improvement of kidney perfusion, the restoration of RAAS pathway also contributes to renal improvement, as animals and patients receiving Ang2 exhibited improved urinary output and creatinine clearance.^6^, ^8^ As for the improvement in lung function, it was shown that the pulmonary vascular endothelium, where angiotensin‐converting enzyme (ACE) is primarily (+90%) located in humans, is essential for the synthesis and degradation of Ang2, and it has been demonstrated that ACE activity is altered in severe lung injury, and this dysfunction correlates with the severity of the disease.^9^, ^10^, ^11^ Finally, it is known that RAAS is involved in pro‐inflammatory and pro‐fibrotic effects at cellular and molecular levels, and therefore the strong effect on inflammation, as an independent driver of morbidity and mortality in shock, was anticipated and already observed in a pre‐clinical study: anti‐DPP3 antibody treated pigs had strongly decreased interleukin‐6 heart expression in comparison to placebo‐treated controls in a septic shock model.^6^

## Conclusions

In this preliminary uncontrolled case series of terminally critically ill patients with refractory shock due to septic shock and septic cardiomyopathy, the administration of the specific inhibitory anti‐DPP3 antibody was well tolerated and followed by improvements in clinical parameters. It is important to note that the non‐controlled design and the small sample size preclude any definitive statements about the safety and potential efficacy of the anti‐DPP3 antibody in refractory shock patients, and that no causality can be claimed between the application of the anti‐DPP3 antibody and the shock reversal observed in the three patients. Still, the results of this case series are encouraging for the ongoing clinical development programme.

## Supporting information


**Supplementary Table S1.** Detailed clinical baseline and follow‐up report of patient 1.

**Supplementary Table S2.** Detailed clinical baseline and follow‐up report of patient 2.

**Supplementary Table S3.** Detailed clinical baseline and follow‐up report of patient 3.
