# Supplementary material for: Targeting dipeptidyl peptidase 3 (DPP3) in extreme‐critically ill patients with refractory shock: First‐in‐human report on the safety and efficacy of an anti‐DPP3 antibody
Source: Eur J Heart Fail. 2025 Jul 10;27(8):1483–5. doi: 10.1002/ejhf.3718 (PMC12482835; doi:10.1002/ejhf.3718)
Supplement: Supplementary file 1 — Supplementary Table S1. Detailed clinical baseline and follow‐up report of patient 1. [file EJHF-27-1483-s003.docx]

**Supplemental table 1**. Detailed clinical baseline and follow-up report of patient 1.

| **Diagnoses at time of treatment with anti-DPP3 antibody** | Refractory shock  Secondary diagnoses:  Pneumogenic sepsis  Severe acute respiratory distress syndrome (ARDS)  Acute myocardial infarction (due to hemodynamic instability revascularization could not be performed)  Acute kidney failure with anuria and lactic acidosis  Disseminated intravascular coagulation  Premorbid history:  Chronic obstructive pulmonary disease (COPD)  Peripheral pulmonary embolism  Arterial hypertension  Obesity  Intermittent atrial flutter  Hypothyroidism |
| --- | --- |
| **Anamnesis** | Female patient of 64 years, weight 110 kg, height 170 cm (body mass index 38 kg/m^2^).  Pre-ICU: Presentation with progressive dyspnea on top of known COPD in peripheral hospital, where she was transferred to intensive care unit on the same day due to rapid clinical deterioration and the requirement of invasive mechanical ventilation.  Transfer to university medical center: on day 3, due to further hemodynamic deterioration and refractory shock.  Rapid deterioration with multiple organ failure including:   - Acute circulatory failure with high norepinephrine requirement and increased lactate - Acute kidney injury with anuria requiring continuous renal replacement therapy. - Respiratory failure requiring invasive mechanical ventilation   ‑Underlying acute myocardial infarction, which could not be treated with revascularization due to hemodynamic instability. |
| **Medication besides anti-DPP3 antibody** | Anti-inflammatory/ anti-infective treatment:  Hydrocortisone  Meropenem  Vancomycin  Levofloxacine  Hemodynamic treatment:  Norepinephrine  Volume  Sedation/ anesthesia:  Propofol  Isoflurane  Sufentanil  Midazolam  Other treatment:  Aspirine  Enoxaparine  Ipratropium/ Salbutamol  Levothroxine  Potassium chloride  Vitamine B1  Vitamine B6  Nutrition  Note: No relevant changes in medication and no introduction of new medication during and after initiation of anti-DPP3 antibody within the time-span of 72 hours. |
| **Independent assessment by patient selection board** | Due to multiple organ failure, the patient prognosis was deemed poor and standard-of-care treatment options exhausted. Expected mortality was calculated as > 90% based on SAPS-3. cDPP3 levels were 125 ng/mL.  In view of the clinical scenario, the patient selection board decided to administer the anti-DPP3 antibody. |
| **Outcome of anti-DPP3 antibody treatment (48 hours)** | Anti-DPP3 antibody was well tolerated, no adverse reactions were observed.  Shock reversal according to predefined criteria achieved (At time-point 48 hours norepinephrine dose ≤0.2µg/kg body weight or halving of initial dose)  Decrease in DPP3 activity in the bloodstream.  Improvement of respiratory function as observed by increase in P/F ratio.  Improvement of renal function as indicated by creatinine.  Normalized lactate.  Reduced inflammation (decrease in CRP and IL6). |
| **Overall outcome** | After initial improvement, patient suffered new clinical deterioration and relapse of circulatory shock, most probably due to untreated myocardial infarction leading to rupture of the papillary muscle followed by fatal mitral valve insufficiency on day 11 after treatment with the anti-DPP3 antibody. |

| **Course of clinical and laboratory parameters before, during and after patient-based treatment approach with anti-DPP3 antibody** | | | | | | | | | | | | | |
| --- | --- | --- | --- | --- | --- | --- | --- | --- | --- | --- | --- | --- | --- |
| **Variable (reference values)** | **Before anti-DPP3 antibody** | **Day 1** | **Day 2** | **Day 3** | **Day 4** | **Day 5** | **Day 6** | **Day 7** | **Day 8** | **Day 9** | **Day 10** | **Day 11/ Death** | **Comment/**  **Clinical interpretation** |
| Dipeptidyl peptidase 3 activity [µmol/min] | 2,33E-05 | 3,10E-06 | 3,83E-06 | 6,29E-06 | 6,95E-06 | n/a | 9,27E-06 | n/a | n/a | n/a | n/a | n/a |  |
| Norepinephrine [µg/kg/min] | 0.800 | 0.320 | 0.349 | 0.437 | 0.313 | 0.218 | 0.436 | 0.516 | 0.564 | 0.560 | 2.330 | 0.589 |  |
| Lactate (≤2 mmol/L) | 3.5 | 0.9 | 0.8 | 0.8 | 0.7 | 0.7 | 0.8 | 1.1 | 0.9 | 0.8 | 1.2 | 9.3 |  |
| P/F ratio [mmHg] | 94.4 | 295 | 228 | 178 | 227 | 229 | 239 | 77.2 | 107 | 143 | 154 | 56.2 |  |
| Interleukin-6 (<4.4 ng/L) | 40.9 | 11.3 | 8.5 | 4.8 | n/a | n/a | n/a | n/a | n/a | n/a | 26.6 | n/a |  |
| C-reactive protein (<5 mg/L) | 298 | 273 | 179 | 99 | 63 | 51 | 43 | 55 | 49 | 42 | 38 | 7 |  |
| Procalcitonine (0.5 µg/L) | 2.33 | n/a | n/a | n/a | 0.67 | n/a | 0.81 | n/a | n/a | n/a | 1.30 | n/a |  |
| Creatinine (0.55-1.02 mg/dL) | 3.26 | 2.28 | 1.67 | 1.42 | 1.35 | 1.38 | 1.61 | 1.60 | 1.42 | 1.37 | 1.41 | 1.12 |  |
| Urea (9.0-23.0 mg/dL) | 25.5 | 29.8 | 35.7 | 38.5 | 39.9 | 45.5 | 49.8 | 55.0 | 49.5 | 49.4 | 53.8 | 23.7 |  |
| GFR (CKD-EPI, [m/min]) | 14 | 22 | 32 | 39 | 41 | 40 | 33 | 34 | 39 | 41 | 39 | 52 |  |
| AST/GOT (<35 U/L) | 123 | 437 | 327 | 200 | 182 | 148 | 139 | 109 | 76 | 69 | 87 | 27376 | Increase due to underlying disease and delayed liver shock |
| ALT/GPT (<35 U/L) | 63 | 284 | 298 | 268 | 255 | 244 | 224 | 191 | 144 | 119 | 110 | 8681 | Increase due to underlying disease and delayed liver shock |
| Gamma-GT (<38U/L) | 137 | 126 | 141 | 207 | 516 | 666 | 614 | 623 | 435 | 345 | 410 | 152 | Increase due to underlying disease and delayed liver shock |
| Total bilirubin (0.3-1.2 mg/dL) | 1.5 | 1.2 | 1.1 | 1.4 | 1.8 | 1.9 | 2.0 | 2.7 | 2.4 | 2.4 | 2.8 | 5.6 |  |
| Lactate dehydrogenase  (120-246 U/L) | n/a | n/a | n/a | 474 | n/a | 83 | n/a | n/a | n/a | n/a | 18641 | 37254 |  |
| Lipase (12-53 U/L) | 28 | 42 | 64 | 74 | 55 | 46 | 60 | 77 | 96 | 104 | 150 | 120 |  |
| Troponin I (<38.64 pg/mL) | 2107 | 2317 | 1211 | 765 | n/a | n/a | n/a | n/a | n/a | n/a | n/a | n/a |  |
| NTproBNP (<125 ng/L) | 52001 | n/a | n/a | n/a | n/a | n/a | n/a | n/a | n/a | n/a | n/a | n/a |  |
| Potassium (3.5-5.0 mmol/L) | 4.6 | 4.4 | 4.6 | 4.3 | 4.3 | 4.3 | 4.5 | 4.2 | 3.7 | 4.4 | 4.3 | 5.5 |  |
| Sodium (135-145 mmol/L) | 139 | 141 | 142 | 142 | 142 | 142 | 142 | 142 | 142 | 141 | 141 | 139 |  |
| Calcium (1.12-1.32 mmol/L) | 1.11 | 1.19 | 1.21 | 1.13 | 1.13 | 1.13 | 1.17 | 1.14 | 1.12 | 1.24 | 1.22 | 0.90 |  |
| Chloride (99-111 mmol/L) | 107 | 107 | 107 | 109 | 110 | 110 | 109 | 107 | 110 | 107 | 107 | 109 |  |
| Glucose (70-105 mg/dL) | 185 | 180 | 180 | 171 | 169 | 169 | 154 | 155 | 152 | 166 | 147 | 202 |  |
| pH (7.36-7.44) | 7.38 | 7.39 | 7.33 | 7.43 | 7.42 | 7.42 | 7.41 | 7.38 | 7.34 | 7.38 | 7.39 | 7.10 |  |
| Base excess (-2.0-3.0 mmol/L) | -3.6 | 1.5 | 1.8 | 2.0 | 0.4 | 0.4 | 0.6 | 0.5 | -1.6 | 0.3 | 0.5 | -13.3 |  |
| Quick (84-129%) | 102 | n/a | n/a | 93 | 82 | 89 | 81 | 99 | 103 | 97 | 76 | 46 |  |
| PTT (26-36 sec) | 34 | n/a | n/a | 28 | 30 | 28 | 30 | 28 | 26 | 28 | 29 | 79 |  |
| Thrombin time (<19.0 sec) | 14.6 | n/a | n/a | 15.8 | 17.1 | 16.3 | 16.7 | 15.7 | 15.9 | 17.8 | 17.7 | 31.7 |  |
| Fibrinogen (1.90-3.70 g/L) | >3.70 | n/a | n/a | >3.70 | >3.70 | >3.70 | >3.70 | >3.70 | >3.70 | 3.37 | 3.09 | n/a |  |
| Hemoglobin (12.4-16.1 g/dL) | 10.8 | 9.5 | 10.0 | 10.3 | 9.5 | 9.7 | 9.4 | 9.4 | 8.7 | 8.1 | 7.6 | 8.6 |  |
| Erythrocytes (4.01-5.29 bn/mL) | 3.32 | 2.92 | 3.07 | 3.14 | 2.97 | 2.98 | 2.91 | 2.78 | 2.51 | 2.38 | 2.18 | 2.65 |  |
| Hematocrit (35-45%) | 32.5 | 28.5 | 30.4 | 31.2 | 30.6 | 31.5 | 30.4 | 29.6 | 26.1 | 25.6 | 24.0 | 24.4 |  |
| MCV (80-95 fl) | 98 | 98 | 99 | 99 | 103 | 106 | 105 | 106 | 104 | 108 | 110 | 92 |  |
| MCH (27.0-32.9 pg) | 32.5 | 32.5 | 32.6 | 32.8 | 32.0 | 32.6 | 32.3 | 33.8 | 34.7 | 34.0 | 34.9 | 32.5 |  |
| MCHC (32.6-36.5 g/dL) | 33.2 | 33.3 | 32.9 | 33.1 | 31.1 | 30.8 | 30.9 | 31.7 | 33.4 | 31.6 | 31.7 | 35.2 |  |
| EVB (11.5-14.5%) | 14.1 | 14.3 | 15.1 | 16.0 | 17.1 | 17.3 | 18.3 | 18.9 | 22.2 | 21.7 | 23.1 | 18.2 |  |
| Leukocytes (4.0-11.8 bn/L) | 17.5 | 14.3 | 18.2 | 20.1 | 24.2 | 28.0 | 27.7 | 25.2 | 28.7 | 23.6 | 26.9 | 11.8 |  |
| Thrombocytes (150-370 bn/L) | 178 | 134 | 151 | 130 | 124 | 126 | 106 | 100 | 85 | 94 | 104 | 74 |  |
