# Supplementary material for: Targeting dipeptidyl peptidase 3 (DPP3) in extreme‐critically ill patients with refractory shock: First‐in‐human report on the safety and efficacy of an anti‐DPP3 antibody
Source: Eur J Heart Fail. 2025 Jul 10;27(8):1483–5. doi: 10.1002/ejhf.3718 (PMC12482835; doi:10.1002/ejhf.3718)
Supplement: Supplementary file 2 — Supplementary Table S2. Detailed clinical baseline and follow‐up report of patient 2. [file EJHF-27-1483-s001.docx]

**Supplemental table 2**. Detailed clinical baseline and follow-up report of patient 2.

| **Diagnoses at time of treatment with anti-DPP3 antibody** | Refractory refractory shock  Secondary diagnoses:  Pneumogenic sepsis with Escherichia coli bacteremia  Septic cardiomyopathy and acute myocardial infarction (AMI)  Severe acute respiratory distress syndrome (ARDS)  Acute kidney injury with anuria and lactic acidosis  Premorbid history:  Coronary heart disease (AMI 8 weeks before anti-DPP3 antibody administration)  Intermittent atrial fibrillation  Arterial hypertension  Hypertensive cardiomyopathy  Aortic valve stenosis  Peripheral artery disease  Cholangitis with choledocholithiasis, subsequent endoscopic papillotomy with stone extraction from the bile duct and post-ERCP (endoscopic retrograde cholangiopancreatography) duodenal perforation 6 weeks before anti-DPP3 antibody administration; Followed by cholecystectomy, and postoperative retroperitoneal abscess. |
| --- | --- |
| **Anamnesis** | Male patient of 84 years, weight 73 kg, height 164 cm (body mass index 27 kg/m^2^).  Hospitalization: The patient´s hospitalization began 6 weeks before the administration of anti-DPP3 antibody. The patient was initially admitted to hospital for cholangitis, underwent ERCP, which was followed by a duodenal perforation, cholecystectomy and postoperative retroperitoneal abscess. Due to these complications, the patient had several stays on ICU.  Final transfer to ICU: deterioration due to cardiac and respiratory decompensation. Further deterioration into refractory shock with multiple organ failure including:   - Acute circulatory failure with highest norepinephrine requirement and massively increased lactate up to 7.1 mmol/L - Acute kidney injury with anuria requiring continuous renal replacement therapy. - Respiratory failure requiring invasive mechanical ventilation. |
| **Medication besides anti-DPP3 antibody** | Anti-inflammatory/ anti-infective treatment:  Hydrocortisone  Piperacillin/ Tazobactam (later switched to Meropenem)  Ciprofloxacin  Anidulafungin  Hemodynamic treatment:  Norepinephrine  Dobutamine  Volume  Sedation/ anesthesia:  Propofol  Sufentanil  Other treatment:  Aspirine  Enoxaparine  Amiodarone  Atorvastatin  Potassium chloride  Magnesium  Vitamine B1  Nutrition  Note: No relevant changes in medication and no introduction of new medication during and after initiation of anti-DPP3 antibody within the time-span of 72 hours. |
| **Independent assessment by patient selection board** | Due to multiple organ failure, the patient prognosis was deemed extremely poor and standard-of-care treatment options exhausted. cDPP3 levels were 89 ng/mL.  In view of the clinical scenario, the patient selection board decided to administer anti-DPP3 antibody. |
| **Outcome after anti-DPP3 antibody treatment (48 hours)** | Anti-DPP3 antibody was well tolerated, no adverse reactions were observed.  Shock reversal according to predefined criteria achieved.  (At time-point 48 hours norepinephrine dose ≤0.2µg/kg/body weight or halving of initial dose).  Decrease in DPP3 activity in the bloodstream.  Improvement of respiratory function as observed by increase in P/F ratio.  Improvement of renal function as indicated by creatinine.  Reduced lactate.  Reduced inflammation (decrease in CRP and IL6). |
| **Overall outcome** | Patient was transferred eight weeks after treatment with anti-DPP3 antibody (AK1967) to rehabilitation clinic. Despite timely shock reversal, transfer was delayed due to prolonged respiratory recovery and acute myocardial infarction (4 weeks after anti-DPP3 antibody; implantation of two drug-eluting stents into left main vessel). Liver and renal function recovered, with renal replacement therapy applied until two weeks after anti-DPP3 antibody administration. |

| **Course of clinical and laboratory parameters before, during and after patient-based treatment approach with anti-DPP3 antibody**  **(censored after day 10 post-treatment, since total hospitalization period is over 60 days)** | | | | | | | | | | | | |
| --- | --- | --- | --- | --- | --- | --- | --- | --- | --- | --- | --- | --- |
| **Variable** | **Before anti-DPP3 antibody** | **Day 1** | **Day 2** | **Day 3** | **Day 4** | **Day 5** | **Day 6** | **Day 7** | **Day 8** | **Day 9** | **Day 10** | **Comment/**  **Clinical interpretation** |
| Dipeptidyl peptidase 3 activity [µmol/min] | 1,76E-05 | 8,03E-06 | 5,72E-06 | 5,19E-06 | n/a | n/a | 3,82E-06 | n/a | n/a | n/a | n/a |  |
| Norepinephrine [µg/kg/min] | 1.42 | 1.26 | 0.384 | 0.427 | 0.274 | 0.082 | 0.055 | 0.007 | 0 | 0 | 0 |  |
| Dobutamine [µg/kg/min] | 2.28 | 2.28 | 3.42 | 2.28 | 0 | 0 | 0 | 0 | 0 | 0 | 0 |  |
| Lactate (≤2 mmol/L) | 7.1 | 6.2 | 2.3 | 1.3 | 1.0 | 1.5 | 1.3 | 1.4 | 1.3 | 1.3 | 1.1 |  |
| P/F ratio [mmHg] | 83.5 | 192 | 182 | 127 | 152 | 229 | 231 | 250 | 249 | 250 | 243 |  |
| Interleukin-6 (<4.4 ng/L) | 241306 | 5016 | 408.0 | 117.7 | n/a | n/a | n/a | n/a | n/a | n/a | n/a |  |
| C-reactive protein (<5 mg/L) | 166 | 331 | 332 | 296 | 211 | 195 | 172 | 135 | 111 | 111 | 91 |  |
| Procalcitonine (0.5 µg/L) | 30.41 | 35.94 | 21.79 | n/a | n/a | n/a | n/a | n/a | n/a | n/a | n/a |  |
| Creatinine (0.70-1.30 mg/dL) | 2.29 | 1.66 | 0.86 | 0.87 | 1.00 | 0.96 | 1.00 | 1.04 | 0.94 | 0.86 | 0.82 |  |
| Urea (9.0-23.0 mg/dL) | 32.8 | 21.4 | 12.9 | 14.4 | 18.4 | 19.1 | 18.2 | 17.8 | 17.8 | 16.3 | 15.7 |  |
| GFR (CKD-EPI, [m/min]) | 25 | 37 | 79 | 79 | 68 | 72 | 69 | 66 | 73 | 79 | 81 |  |
| AST/GOT (<50 U/L) | 52 | 616 | 386 | 219 | 113 | 95 | 65 | 58 | 38 | 34 | 34 | Increase due to underlying disease and delayed liver shock |
| ALT/GPT (<50 U/L) | 30 | 232 | 185 | 147 | 109 | 79 | 62 | 53 | 38 | 34 | 30 | Increase due to underlying disease and delayed liver shock |
| Glutate dehydrogenase (<7 U/L) | n/a | 251 | 173 | 102 | 59 | 41 | 31 | 20 | 12 | 10 | 11 | Increase due to underlying disease and delayed liver shock |
| Gamma-GT (<73 U/L) | n/a | 206 | 247 | 663 | 775 | 720 | 588 | 464 | 356 | 339 | 333 | Increase due to underlying disease and delayed liver shock |
| Total bilirubin (0.3-1.2 mg/dL) | 0.3 | 0.4 | 0.7 | 0.8 | 0.7 | 0.4 | 0.4 | 0.3 | 0.3 | 0.2 | 0.2 |  |
| Lipase (12-53 U/L) | 36 | 33 | 20 | 21 | 34 | 64 | 64 | 66 | 43 | 42 | 46 |  |
| Troponin I (<53.53 pg/mL) | 1516 | 2229 | 4476 | 3450 | n/a | n/a | n/a | n/a | n/a | n/a | n/a |  |
| Potassium (3.5-5.0 mmol/L) | 4.9 | 3.7 | 4.1 | 4.3 | 3.1 | 4.3 | 4.2 | 4.0 | 4.2 | 4.3 | 4.5 |  |
| Sodium (135-145 mmol/L) | 146 | 143 | 146 | 1.44 | 143 | 141 | 142 | 142 | 141 | 139 | 140 |  |
| Calcium (1.12-1.32 mmol/L) | 1.14 | 1.19 | 1.16 | 1.25 | n/a | 1.23 | 1.13 | 1.19 | 1.12 | 1.16 | 1.17 |  |
| Chloride (99-111 mmol/L) | 112 | 108 | 110 | 109 | 105 | 110 | 110 | 109 | 109 | 109 | 108 |  |
| Glucose (70-105 mg/dL) | 87 | 126 | 156 | 176 | 139 | 144 | 127 | 110 | 150 | 133 | 111 |  |
| pH (7.36-7.44) | 7.24 | 7.28 | 7.44 | 7.36 | 7.39 | 7.49 | 7.44 | 7.46 | 7.45 | 7.44 | 7.41 |  |
| Base excess (-2.0-3.0 mmol/L) | -6 | -4 | 4.3 | 3.4 | 0.4 | 3.1 | 2.7 | 5.1 | 3.7 | 1.6 | 1.5 |  |
| Quick (84-129%) | 85 | n/a | n/a | 108 | n/a | 104 | n/a | n/a | 94 | n/a | 89 |  |
| PTT (26-36 sec) | 34 | n/a | n/a | 39 | n/a | 38 | n/a | n/a | 37 | n/a | 35 |  |
| Thrombin time (<19.0 sec) | 17.6 | n/a | n/a | 16.4 | n/a | 16.6 | n/a | n/a | 17.0 | n/a | 17.0 |  |
| Fibrinogen (1.90-3.70 g/L) | >3.70 | n/a | n/a | >3.70 | n/a | >3.70 | n/a | n/a | >3.70 | n/a | >3.70 |  |
| Hemoglobin (13.9-17.7 g/dL) | 8.2 | 10.0 | 8.9 | 9.2 | 9.2 | 9.0 | 8.8 | 8.9 | 8.5 | 8.4 | 7.9 |  |
| Erythrocytes (4.57-5.98 bn/mL) | 2.69 | 3.29 | 2.93 | 2.99 | 3.06 | 2.99 | 2.99 | 2.96 | 2.89 | 2.78 | 2.73 |  |
| Hematocrit (36-48%) | 27.8 | 33.2 | 28.7 | 28.9 | 28.8 | 27.8 | 28.0 | 27.2 | 26.7 | 26.0 | 26.2 |  |
| MCV (80-95 fl) | 103 | 101 | 98 | 96 | 94 | 93 | 94 | 92 | 92 | 93 | 96 |  |
| MCH (27.6-33.2 pg) | 30.5 | 30.4 | 30.4 | 30.8 | 30.1 | 30.1 | 29.4 | 30..1 | 29.4 | 30.2 | 28.9 |  |
| MCHC (33.0-37.2 g/dL) | 29.5 | 30.2 | 31.0 | 31.9 | 31.9 | 32.4 | 31.5 | 32.7 | 31.8 | 32.4 | 30.2 |  |
| EVB (11.5-14.5%) | 17.1 | 17.3 | 17.3 | 16.9 | 16.6 | 16.8 | 16.5 | 16.4 | 16.2 | 16.3 | 16.3 |  |
| Leukocytes (3.8-10.3 bn/L) | 5.0 | 22.1 | 33.3 | 37.5 | 23.6 | 15.2 | 14.9 | 18.3 | 24.5 | 19.1 | 18.1 |  |
| Thrombocytes (150-370 bn/L) | 598 | 410 | 333 | 284 | 214 | 193 | 186 | 225 | 295 | 355 | 440 |  |
