# Supplementary material for: Targeting dipeptidyl peptidase 3 (DPP3) in extreme‐critically ill patients with refractory shock: First‐in‐human report on the safety and efficacy of an anti‐DPP3 antibody
Source: Eur J Heart Fail. 2025 Jul 10;27(8):1483–5. doi: 10.1002/ejhf.3718 (PMC12482835; doi:10.1002/ejhf.3718)
Supplement: Supplementary file 3 — Supplementary Table S3. Detailed clinical baseline and follow‐up report of patient 3. [file EJHF-27-1483-s002.docx]

**Supplemental table 3**. Detailed clinical baseline and follow-up report of patient 3.

| **Diagnoses at time of treatment with anti-DPP3 antibody** | Refractory shock  Secondary diagnoses:  Septic shock  Pseudomonas aeruginosa bacteremic pyelonephritis  Right-heart decompensation  Acute kidney failure with anuria  Premorbid history:  Endometrioid adenocarcinoma  Atrial fibrillation  Sick-sinus-syndrome and pacemaker implantation in 2019  Tricuspid valve insufficiency  Arterial hypertension  Obesity  Liver cirrhosis  Obstructive sleep apnea syndrome |
| --- | --- |
| **Anamnesis** | Female of 80 years, weight 101 kg, height 169 cm, body-mass-index 35 kg/m^2^.  Pre-ICU: Inpatient admission primarily for laparoscopic hysterectomy/adenectomy. After surgery, patient required 4 days of hemodynamic support on the post-anesthesia care unit. Transfer to normal ward on day 4 after surgery.  Transfer to ICU: on day 6 due to suspected septic shock with reduced vigilance and hypotension. Concomitant diagnosis of severe right heart decompensation and respective cardiogenic shock.  Rapid clinical deterioration with multiple organ failure including:   - Acute circulatory failure with high norepinephrine requirement up to 0.55 ug/kg/min and increased lactate up to 7.2 mmol/L - Acute kidney injury with anuria requiring continuous renal replacement therapy. - Respiratory failure requiring invasive mechanical ventilation |
| **Medication besides anti-DPP3 antibody** | Anti-inflammatory/ anti-infective treatment:  Hydrocortisone  Meropenem  Vancomycin  Ciprofloxacin  Hemodynamic treatment:  Norepinephrine  Milrinone  Volume  Other treatment:  Amiodarone  Potassium  Furosemide  Magnesium  Haloperidol  Melatonine  Insulin  Vitamine B1  Nutrition  Note: No relevant changes in medication and no introduction of new medication during and after initiation of anti-DPP3 antibody within the time-span of 72 hours. |
| **Independent assessment by patient selection board** | Due to multiple organ failure, the patient prognosis was deemed extremely poor and standard-of-care treatment options exhausted. cDPP3 levels were 57 ng/mL.  In view of the clinical scenario, the patient selection board decided to administer anti-DPP3 antibody. |
| **Outcome anti-DPP3 antibody treatment (48 hours)** | Anti-DPP3 antibody was well tolerated, no adverse reactions were observed.  Shock reversal according to predefined criteria achieved (At time-point 48 hours norepinephrine dose ≤0.2µg/kg/body weight or halving of initial dose)  Decrease in DPP3 activity in the bloodstream.  Improvement of respiratory function as observed by increase in P/F ratio.  Improvement of renal function as indicated by creatinine.  Normalized lactate.  Reduced inflammation (decrease in CRP and IL6). |
| **Overall outcome** | After initial improvement allowing for mobilization and to plan transfer to peripheral ward, exacerbation of right heart failure (possibly due to positive fluid balance) occurred, leading to refractory shock and death on day 4 after treatment with anti-DPP3 antibody. |

| **Course of clinical and laboratory parameters before, during and after patient-based treatment approach with anti-DPP3 antibody** | | | | | | |
| --- | --- | --- | --- | --- | --- | --- |
| **Variable** | **Before anti-DPP3 antibody** | **Day 1** | **Day 2** | **Day 3** | **Day 4/ Death** | **Comment/**  **Clinical interpretation** |
| Dipeptidyl peptidase 3 activity [µmol/min] | 1,47E-05 | 8,34E-06 | 9,00E-06 | 9,79E-06 | n/a |  |
| Norepinephrine [µg/kg/min] | 0.554 | 0.396 | 0.119 | 0.139 | 0.376 | In the evening of day 4 after anti-DPP3 antibody continuous increase in dose to 1.98 µg/kg/min in the process of dying |
| Milrinone [µg/kg/min] | 0.165 | 0.165 | 0 | 0 | 0.165 |  |
| Lactate (≤2.0 mmol/L) | 7.2 | 3.6 | 1.7 | 2.7 | 3.7 | In the evening of day 4 after continuous increase to 13 mmol/L in the process of dying |
| P/F ratio [mmHg] | 176 | 211 | 257 | 217 | 296 |  |
| Interleukin-6 (<4.4 ng/L) | 893.5 | 67.8 | 27.2 | n/a | n/a |  |
| C-reactive protein (<5 mg/L) | 341 | 351 | 252 | 132 | 97 |  |
| Procalcitonine (0.5 µg/L) | 42.94 | 31.00 | 18.03 | n/a | 5.44 |  |
| Creatinine (0.55-1.02 mg/dL) | 1.53 | 0.91 | 0.74 | 0.77 | 0.94 |  |
| Urea (9.0-23.0 mg/dL) | 19.1 | 13.8 | 20.1 | 28.0 | 38.8 |  |
| GFR (CKD-EPI, [m/min]) | 32 | 60 | 77 | 73 | 57 |  |
| AST/GOT (<35 U/L) | 81 | 120 | 97 | 74 | 114 | Mild increase interpreted as an expression of the underlying disease and delayed liver shock |
| ALT/GPT (<35 U/L) | 42 | 68 | 53 | 49 | 42 | Mild increase interpreted as an expression of the underlying disease and delayed liver shock |
| GLDH (<5 U/L) | 5 | 30 | 17 | 10 | 14 | Mild increase interpreted as an expression of the underlying disease and delayed liver shock |
| Gamma-GT (<38U/L) | 112 | 102 | 102 | 141 | 183 | Mild increase interpreted as an expression of the underlying disease and delayed liver shock |
| Total bilirubin (0.3-1.2 mg/dL) | 1.1 | 1.8 | 1.9 | 1.8 | 4.4 | Mild increase interpreted as an expression of the underlying disease and delayed liver shock |
| Lipase (12-53 U/L) | 20 | 18 | 28 | 37 | n/a |  |
| Troponin I (<38.64 pg/mL) | n/a | n/a | n/a | n/a | 15 |  |
| NTproBNP (<450 ng/L) | n/a | n/a | n/a | n/a | 8825 | Severe right-heart decompensation, as also evidenced by initial echocardiography |
| Potassium (3.5-5.0 mmol/L) | 4.2 | 4.2 | 4.3 | 4 | 4.5 |  |
| Sodium (135-145 mmol/L) | 142 | 144 | 143 | 142 | 141 |  |
| Calcium (1.12-1.32 mmol/L) | 1.33 | 1.15 | 1.19 | 1.3 | 1.06 |  |
| Chloride (99-111 mmol/L) | 106 | 106 | 105 | 103 | 104 |  |
| Glucose (70-105 mg/dL) | 137 | 207 | 147 | 192 | 146 |  |
| pH (7.36-7.44) | 7.46 | 7.49 | 7.44 | 7.47 | 7.45 |  |
| Base excess (-2.0-3.0 mmol/L) | -1.5 | 4.8 | 5.9 | 7.5 | 2.6 |  |
| Quick (84-129%) | 46 | 48 | 62 | 55 | 41 |  |
| PTT (26-36 sec) | 42 | 42 | 38 | 32 | 36 |  |
| Thrombin time (<19.0 sec) | 16.9 | 17.0 | 19.7 | 21.3 | 24.0 |  |
| Fibrinogen (1.90-3.70 g/L) | >3.70 | >3.70 | >3.70 | >3.70 | 3.57 |  |
| Hemoglobin (12.4-16.1 g/dL) | 9.8 | 9.3 | 9.0 | 9.2 | 9.0 |  |
| Erythrocytes (4.01-5.29 bn/mL) | 3.04 | 2.87 | 2.94 | 3.04 | 2.90 |  |
| Hematocrit (35-45%) | 28.6 | 27.1 | 27.2 | 28.4 | 26.5 |  |
| MCV (80-95 fl) | 94 | 94 | 93 | 93 | 91 |  |
| MCH (27.0-32.9 pg) | 32.2 | 32.4 | 30.6 | 30.3 | 31.0 |  |
| MCHC (32.6-36.5 g/dL) | 34.3 | 34.3 | 33.1 | 32.4 | 34.0 |  |
| EVB (11.5-14.5%) | 16.2 | 16.4 | 16.7 | 16.7 | 17.8 |  |
| Leukocytes (4.0-11.8 bn/L) | 35.8 | 63.0 | 55.0 | 41.4 | 46.2 |  |
| Thrombocytes (150-370 bn/L) | 113 | 115 | 92 | 61 | 50 |  |
